# SELLA TURCICA 3T MAGNETIC RESONANCE IMAGING IN THE DIAGNOSIS OF CUSHING’S DISEASE IN CHILDREN: TWO CASE REPORTS

**DOI:** 10.1590/1984-0462/;2019;37;3;00009

**Published:** 2019-05-09

**Authors:** Leila Warszawski, Gabriel Santi Calabria Esteves, Ariane Pagnocelli, Bruna de Lacerda Bouzon, Sayra Lacerda de Oliveira

**Affiliations:** a Instituto Estadual de Diabetes e Endocrinologia Luis Capriglione, Rio de Janeiro, RJ, Brazil.

**Keywords:** Pituitary gland, Adenoma, Cushing syndrome, Child, Magnetic resonance, Hipófise, Adenoma, Síndrome de Cushing, Criança, Ressonância magnética

## Abstract

**Objective:**

To present two clinical cases of pediatric Cushing disease caused by adrenocorticotropic hormone secreting pituitary adenomas, which were diagnosed by magnetic resonance imaging using 3 Tesla technology.

**Case description:**

Two cases of Cushing disease in 9-year-old children are reported. Both children presented pituitary adenomas that were smaller than 5 mm at their largest diameter, and which were not seen by standard 1.5 Tesla resonance. One of the patients was submitted to bilateral and simultaneous catheterization of the inferior petrosal sinus, but the result was undetermined. In both cases, the pituitary adenoma was detected by 3 Tesla magnetic resonance imaging. Both patients underwent transsphenoidal surgery and were cured.

**Comments:**

Cushing disease presents high morbidity. Therefore, early diagnosis and prompt treatment are essential. It is usually caused by adenomas that are smaller than 5 mm in diameter. Surgery is the first line of treatment, and effective methods of locating the adenoma are necessary for greater therapeutic success. This report suggests that the 3 Tesla magnetic resonance imaging is more sensitive, and thus able to detect pituitary microadenomas (largest diameter <10 mm). This exam may be indicated as a low-morbidity diagnostic tool for finding pituitary microadenomas in Cushing disease that are not visualized by 1.5 Tesla magnetic resonance imaging.

## RESUMO

**Objetivo:**

Apresentar dois casos clínicos de Doença de Cushing infantil decorrentes de adenoma hipofisário secretor de hormônio adrenocorticotrófico, cujo diagnóstico foi realizado por meio da ressonância magnética pela tecnologia 3 Tesla.

**Descrição do caso:**

São relatados dois casos de Doença de Cushing em crianças aos nove anos. Ambas apresentavam adenomas menores que 5 mm em seu maior diâmetro que não foram visualizados por meio de ressonância magnética de sela turca utilizando tecnologia 1,5 Tesla. Uma das pacientes foi submetida ao cateterismo bilateral e simultâneo do seio petroso inferior, porém com resultado indeterminado. Nas duas, o adenoma hipofisário foi visualizado mediante ressonância magnética utilizando tecnologia 3 Tesla. Ambas foram submetidas à cirurgia transesfenoidal e evoluíram para cura.

**Comentários:**

A Doença de Cushing apresenta alta morbidade, necessitando de diagnóstico e tratamento precoces, e geralmente é causada por adenomas com diâmetro inferior a 5 mm. O tratamento é cirúrgico, sendo preciso utilizar métodos eficazes de localização do adenoma para maior sucesso terapêutico. Esses relatos sugerem que a ressonância magnética 3 Tesla tem mais sensibilidade na detecção de microadenomas hipofisários (maior diâmetro <10 mm), podendo-se indicar esse exame como uma ferramenta diagnóstica de baixa morbidade na localização de microadenomas hipofisários na Doença de Cushing não visualizados pela ressonância magnética 1,5 Tesla.

## INTRODUCTION

Cushing’s syndrome occurs from exposure to supra-physiological levels of glucocorticoids. ^1^ ^,^ ^2^ ^,^ ^3^ ^,^ ^4^ ^,^ ^5^ In childhood and adolescence, the most frequent clinical findings are: generalized obesity associated with growth retardation, and delayed bone age. ^6^ ^,^ ^7^ ^,^ ^8^ ^,^ ^9^ Other less specific manifestations are pubertal retardation, fatigue, acne, depression, hypertension and hirsutism. ^6^ ^,^ ^7^ ^,^ ^8^ ^,^ ^9^

Stretch marks and a hunched back, which are characteristic in adulthood, are often absent. The symptoms initially are not taken seriously. The mean time between the clinical manifestations and the diagnosis is two years. ^1^ Cushing’s syndrome presents high morbidity in children and adults, requiring early diagnosis and treatment. ^1^ ^,^ ^2^ ^,^ ^3^ ^,^ ^4^ ^,^ ^5^ ^,^ ^6^ ^,^ ^7^ ^,^ ^8^ ^,^ ^9^

When Cushing’s Syndrome is suspected, the chronic use of glucocorticoids should be avoided. When it is confirmed that endogenous Cushing’s Syndrome is present, serum levels of an adrenocorticotropic hormone (ACTH) indicate the etiology: <10 pg/mL demonstrate an independent cause, between 10 and 29 pg/mL is considered to be suspicious, while levels >30 pg/mL and especially >50 pg/mL suggest ACTH dependent causes. ^7^ However, levels that are higher than 20 pg/mL may also indicate ACTH-dependent Cushing’s syndrome. ^1^ ^,^ ^2^ ^,^ ^3^ ^,^ ^4^ ^,^ ^5^ In childhood, Cushing’s disease, a pituitary adenoma producer of ACTH, is the most common etiology of Cushing Syndrome over the age of 5.^1^ ^,^ ^6^ ^,^ ^7^ ^,^ ^8^ ^,^ ^9^ However, ectopic ACTH secretion is very rare (<1‒3%) in children and adolescents.^6^ ^,^ ^7^ ^,^ ^8^

Cushing’s disease mainly occurs with micro-adenoma (largest diameter <10 mm), and an average diameter of 5 mm.^1^ ^,^ ^4^ ^,^ ^7^ ^,^ ^10^ People with Cushing’s disease are recommended to have surgery, and the preoperative localization of the adenoma allows for greater surgical success. ^1^ ^,^ ^2^ ^,^ ^3^ ^,^ ^4^ ^,^ ^5^ The magnetic resonance imaging (1.5T MRI) with 1.5 Tesla technology, which is conventionally used, has low sensitivity when detecting these small adenomas. Magnetic resonance imaging (MRI) using 3 Tesla (RM 3T) technology offers a higher magnetic field, resulting in finer cuts, better image quality and a greater ability to differentiate between adenoma and normal pituitary tissue. ^10^ ^,^ ^11^ ^,^ ^12^ ^,^ ^13^ 3T MRI has been studied with regard to the detection of small adenomas, like those present from Cushing’s Disease.

The purpose of this report is to describe two cases of Cushing’s Disease in children, whose 3T MRIs detected adenomas that were not seen by the 1.5T MRI.

## CASE REPORT

Patients received follow up care at the pediatric endocrinology outpatient clinic. For this report, we used retrospective information obtained through a thorough analysis of their medical records. The study was submitted and approved by the Research Ethics Committee of the hospital.

### Case 1

Black female patient; nine years and three months old. She had progressive weight gain and a decrease in growth velocity, which had been observed since the age of 5 years old. Height1.14 m, Z score -3.24 (below the percentile <2.5); weight 37 kg, Z score 1.33 (>P97); Body Mass Index (BMI) 28.6 kg/m^2^, Z score 3.2 (>P97); acanthosis nigricans, hypertrichosis, hunch back, globular abdomen without purplish stretch marks. Developmental stage in puberty according to the Tanner Scale M3 P5. Bone age of six years and ten months old, with a chronological age of nine years and three months old. No history of glucocorticoid use.

**Figure 1:**
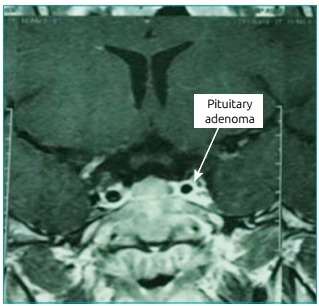
Magnetic Resonance Imaging of Turcica 3 TESLA: Dynamic study demonstrating 2.9mm adenoma, T1-weighted coronal cut of contrast to the left (gadolinium), in the patient from Case 1.

Laboratory tests (Table 1) showed that she had ­ACTH-dependent hypercortisolism and no apparent pituitary adenoma in three 1.5 MRI studies. Additionally, the patient participated in a dynamic study when she was 9, 10 and 12 years old. Bilateral inferior petrosal sinus sampling (BIPSS) was indicated. The exam was performed three years after the first consultation, and the result was unclear due to technical problems. At 12 years of age, the patient was submitted to a 3-RM MRI in the dynamic study. In the post-contrast (gadolinium) sequence, the sagittal section, and the T1 sequence, a contrasting hypocaptive image was found. It was located in the adenohypophysis on the left side’s cavernous sinus. It measured 2.9 × 2.6 × 2.5 mm, which is compatible with microadenoma (Figure 1). Details of this technique can be found in Table 2. A transsphenoidal surgery was performed on the patient when she was 12 years and 11 months old and because she had enough adrenal to be diagnosed with hypercortisolim, she took prednisone for two years. Even after she was cured of hipercortisolism, she did not grow enough. Recombinant growth hormones were then prescribed, even though the growth hormone test was responsive after glucagon stimulation (Table 1). She thus became officially defined as having idiopathic short stature. The patient’s final height was 1.41 m, which did not reach the family target of 1.61 m.

**Table 1:** Patient exams from Case 1.

Exams	Diagnosis	3 months post-surgery	1 year post-surgery	Reference value
Glucose	98 mg/dL		69 mg/dL	Up to 100 mg/dL
Basal Cortisol 8 hours	31.96 µg/dL	<0.3 µg/dL	12 µg/dL	4.3-22.4 µg/dL
IGF-1	545 ng/mL	266 ng/mL	385 ng/mL	190-805 ng/mL
Urinary free cortisol	615.7 mcg/24 h			28.5-213.7 mcg/24 h
Serum Cortisol after 2 days 8 mg/day	Basal: 19 µg/dL After: 1.7 µg/dL FI 91%			FIr >90% -suggestive CD
ACTH	48.8 pg/mL			Up to 46 pg/mL
GH Post Glucagon		Peak 11.9 mU/L		>5 mU/L

IGF-I: *insulin growth factor* I Dexa: dexamethasone; FI: Fall index; FIr: Fall index reference value; CD: Cushing’s disease; ACTH: Adrenocorticotropic hormone; GH: Growth Hormone.

**Table 2:** Features of the 3 Tesla magnetic resonance imaging.

	Case 1	Case 2
Device	Siemens	Siemens
Software	Trio Tim MR B15	Trio Tim MR B15
Post-contrast	3D MP RAGE	TSE
Repetition time	2,530 ms	450 ms
Inversion time	1,100 ms	110 ms
Echo time	3.5 ms	12 ms
Field of vision	25 cm	16 cm
Matrix	512 × 512	448 × 640
Relative bandwidth	190 HZ	195 HZ
Technique	Dynamic	Dynamic
Thickness	2-3 mm	2-3 mm

3D MP RAGE: magnetization prepared rapid acquisition ; TSE: turbo spin echo.

### Case 2

White female patient; nine years and nine months old. She had been overweight since the age of six, and her mother noticed a decrease in growth one year before the consultation. She also exhibited depression and anxiety. She used prednisone until she was seven years old because of bronchial asthma. Height 1.34 m, Z score -0.49 (=P31); weight 59 kg, Z score 3.18 (>P97); BMI 32.9 kg/m^2^, Z score 3.54 (=P99); Moon facies, Tanner development stage of M2 P3, generalized obesity with fat deposition in supraclavicular fossa, cervical acanthosis nigricans, fine axillary hairs, thin purplish stretch marks in abdomen and breasts. Bone age of a nine or ten-year-old, with a chronological age of nine years and six months old.

ACTH-dependent hypercortisolism was confirmed (Table 3). The 1.5T MRI with a dynamic study did not demonstrate the presence of adenoma at the age of 9 years and 10 months, but in the 3T MRI with a dynamic study performed at age 10 years and 3 months, the sagittal cut in the T1 TSE sequence after the administration of (gadolinium) contrast, demonstrated the contrast-enhanced, oval-shaped image of the anterior adiphyophysis measuring 3 × 2.3 × 2.1 mm. Details of the examination technique are described in Table 3. The patient underwent transsphenoidal surgery at ten years of age. She continued to grow with transient diabetes insidious and central permanent hypothyroidism. She presented insufficient adrenal in the postoperative period, which is compatible with the cure for hypercortisolism. She used prednisone for two years. She showed progressive weight loss. Her final height was 1.51 m, with a family target of 1.59 m. She presented new weight gain without the recurrence of hypercortisolism.

**Table 3:** Patient exams from Case 2.

	Diagnostic Hospital 1	Diagnostic Hospital 2	3 months post-surgery	1 year post-surgery	Reference value
Cortisol after 1 mg dexa	5.7 mcg/dL			0.3 mcg/dL	1.8 mcg/dL
Nocturnal Salivary Cortisol	8.3 nmol/L	13.8 nmol/L			<3.6 nmol/L
Urinary Free Cortisol	407.4 mcg/24 h	125.7 mcg/24 h			2-27 mcg/24 h
ACTH	15.7 pg/mL	20 pg/mL	<5 pg/mL		Up to 46 pg/mL
IGF-I	491 ng/mL			428 ng/mL	(79-388 ng/mL) (111-551 ng/mL)
Serum Cortisol after nocturnal suppression 8 mg dexa	Basal: 18.5 µg/dL After: 1.82 µg/dL FI 90.5%				IQ>90% sugestivo DC
Basal cortisol	18.5 mcg/dL		0.5 mcg/dL	1.0 mcg/dL	5-25 mcg/dL
Free T4			0.77 mmol		0.8-1,7 mmol
TSH			7.01 um/L		0.4-5um/L
Basal glycemia/ after 2h	102/180				basal 100 mg/dL 2 Hours up to 140 mg/dL

Dexa: dexamethasone; ACTH: Adrenocorticotropic hormone; IGF-I: *Insulin growth factor* I IQ: Fall index; : CD: Cushing’s disease; TSH: thyroid hormone stimulator.

## DISCUSSION

Cushing’s syndrome in the pediatric population is a rare but serious condition, as chronic exposure to excess glucocorticoids leads to an increased risk of cardiovascular disease, diabetes mellitus, infections, and decreased final height. ^1^ ^,^ ^2^ ^,^ ^6^ ^,^ ^7^ ^,^ ^8^ ^,^ ^9^ Prolonged sickness contributes to increased morbidity and mortality in children and adults. ^3^ In childhood, there may be a period of more than two years between the beginning of the clinical manifestations and the diagnosis, since the non-specific manifestations of this age group may lead to doctors not suspecting the disease. Therefore, care should be taken to allow for early diagnosis and treatment.^6^ ^,^ ^7^ ^,^ ^8^

In the cases described, the patients presented obesity unaccompanied by an increase in food intake and a decrease in growth rate, which is frequent in children with Cushing’s Syndrome. Exogenous obesity does not lead to a decrease in growth velocity.^6^ ^,^ ^7^ ^,^ ^8^ The patient in case 1 had more height impairment, possibly due to the longer time the disease had to evolve before the diagnosis was made. The patient in case 2 used prednisone for bronchial asthma until the age of seven, which may have delayed the clinical suspicion of endogenous Cushing’s Syndrome and caused the fine violet stretch marks.

The precise detection of the etiology avoids unnecessary interventions after the laboratory has confirmed that the disease is endogenous Cushing’s Syndrome. Cushing’s disease is the major etiology of Cushing’s Syndrome for patients over five years of age, while ectopic ACTH secretion is extremely rare in children and adolescents.^6^ ^,^ ^7^ ^,^ ^8^ ^,^ ^9^ Our patients had ACTH-dependent Cushing’s Syndrome, and were therefore submitted to sella túrcica magnetic resonance imaging^1^ ^,^ ^2^ ^,^ ^3^ ^,^ ^4^ ^,^ ^5^. The 1.5T MRI did not show adenomas, which were only diagnosed by means of the 3T MRI. Cushing’s disease usually shows signs of microadenomas, and 50% of them present an average diameter of 5.6 mm, for example, as in our patients, whose largest microadenomas had 3 and 2.9 mm-sized diameters. Sensitivity in the detection of these adenomas by means of the 1.5T MRI, a conventionally used technique, is 50-60%, and computerized tomography is still inferior (40-50%). ^1^ ^,^ ^3^ New techniques with greater visualization sensitivity of microadenomas that have small dimensions are still being evaluated.

The superior magnetic field of the 3T MRI compared to the 1.5T MRI offers greater spatial resolution, which then produces images with a higher resolution and quality, a reduction in the time of acquisition, higher impregnation with (gadolinium) contrast. Furthermore, it minimizes artifacts in the cellar and parassellar regions, which are often associated with thinner cuts (2-3 mm).^10^ ^,^ ^12^ These properties are of fundamental importance in order to differentiate between normal tissue and the adenoma, which then leads to better detection and location of small, frequent microadenomas in Cushing’s disease.^9^ ^,^ ^10^ ^,^ ^11^ ^,^ ^12^ The 3T MRI is useful even when the image is viewed by means of the 1.5T MRI, as it allows for the precise location to be determined in addition to the spatial definition of the adenoma and its relationship to other structures like the cavernous sinus. Furthermore, it helps predict the invasion from adjacent tissue, allowing for better surgical planning.^9^ ^,^ ^10^ ^,^ ^11^ ^,^ ^12^

Some patient series have described the use of 3T MRI in patients with Cushing’s disease. In 19 patients with Cushing’s disease, the 1.5T MRI viewed the adenoma in 12. Four cases were located and one was more well defined through the 3T MRI in relation to the 1.5T MRI.^10^ In four of the six cases in which the 3T MRI did not find anything, there was no cure for the patient after surgery.^10^ In another series with five patients, the 3T MRI provided details of the adenoma in two cases, correcting the lateralization on one of them.^11^ There were also reports of an 11-year-old patient, who had an adenoma detected through the 3T MRI, and not through the 1.5T MRI, which was analogous to the cases described in this publication.^11^ Thus, although there are few studies with a small numbers of patients, the 3T MRI appears to be better than the 1.5T MRI in identifying pituitary microadenomas in Cushing’s disease, both in adults and in children.^10^ ^,^ ^11^ ^,^ ^12^ ^,^ ^13^ As such, it is another tool to diagnose Cushing’s disease. In the cases described, pituitary adenomas with a diameter of less than 5 mm were not detected by the 1.5T MRI but rather, by the 3T MRI. Seeing the adenoma in our patients allowed for surgery to be suggested and the patients to be cured, reinforcing the use of this technology as a diagnostic tool for locating small pituitary adenomas.

It is worth mentioning that the CBSPIS examination is the gold standard examination in differentiating between pituitary and ectopic ACTH secretion as a cause of Cushing’s Syndrome.^1^ ^,^ ^2^ ^,^ ^3^ ^,^ ^4^ ^,^ ^5^ ^,^ ^13^ This examination can also be used for the lateralization of the pituitary adenoma, however, it is an invasive diagnostic method, which can lead to complications such as thrombosis of the lower petrosal breasts. This is of extreme importance for the patients with Cushing’s Syndrome who are in a prothombotic state.^1^ ^,^ ^2^ In addition, there may be indeterminate results, mainly with regard to the lateralization of the adenoma, because of technical errors, such as positioning the catheter the wrong way, lack of experience, and anatomical variation,^11^ ^,^ ^13^ making it so that other complementary methods are required to find and laterize the pituitary adenoma. The patient of case 1 was submitted to this procedure after years of waiting, but had an indeterminate result, possibly due to technical difficulties. The delay in performing this test and its indeterminate result led the patient to be exposed for a longer time to hypercortisolism, possibly contributing to her final height impairment.

The 3T MRI is more accurate than the 1.5T MRI in defining the presence and location of the pituitary microadenoma, often preventing the patient from being submitted to BIPSS. ^10^ ^,^ ^11^ In our patients, the 3T MRI’s detection of the adenoma allowed for surgery to be suggested, even for the patient in which the BIPSS presented indeterminate results. Thus, the patients were cured. Currently, it is established that in patients with ACTH-dependent Cushing’s syndrome, the presence of a pituitary adenoma with a diameter between 6 mm^3^ and 10 mm^3^ (macroadenoma) is highly suggestive of Cushing’s Disease, ^4^ making additional exams like BIPSS unnecessary. These criteria are not well established for the pediatric population.

**Figure 2:**
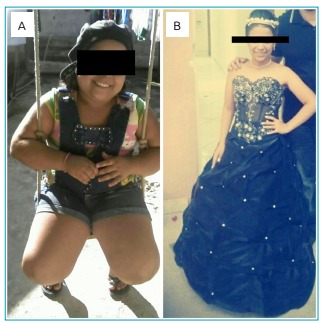
(A) Patient with Cushing’s syndrome phenotype; (B) after surgical curing (Case 1).

The dexamethasone high dose cortisol suppression test, also called Liddle II, although it is seldom used, is useful in differentiating the causes of ACTH-dependent Cushing’s syndrome. The decrease in serum or urinary cortisol above 50% in relation to the baseline is indicative of Cushing’s Disease, even without imaging in the sella túrcica.^14^ ^.^ ^15^ Our patients had cortisol suppression that was greater than 90% in relation to the baseline from the Liddle II test. Being able to see the adenoma made the suggestion of surgery safer, besides it allowed for better surgical planning, possibly contributing to the cure, as clinically demonstrated in Figure 2.

3T MRIs, however, still present little use in clinical practice. The frequency of artifacts that could lead to false-positive results with this technique are not established. Despite this, 3T MRIs may be an additional tool in the management of Cushing’s Disease, which remains a diagnostic and therapeutic challenge. In this context, 7 Tesla magnetic resonance imaging has been studied in the improvement of detection and characterization of pituitary adenomas, in addition to the decrease of artifacts.^13^
